# Frailty and spatialization of older adults in the city of Uberlândia with IVCF-20

**DOI:** 10.11606/s1518-8787.2023057005273

**Published:** 2024-04-01

**Authors:** Rubia Pereira Barra, Edgar Nunes de Moraes, Maria Margaret de Vasconcellos Lemos, Poliana Castro de Resende Bonati, José Flávio Morais Castro, André Augusto Jardim

**Affiliations:** I Centro Colaborador Planificação da Atenção à Saúde Uberlândia Conselho Nacional de Secretários de Saúde Uberlândia MG Brazil Centro Colaborador Planificação da Atenção à Saúde Uberlândia. Conselho Nacional de Secretários de Saúde. Uberlândia, MG, Brazil; II Universidade Federal de Minas Gerais Faculdade de Medicina Departamento de Clínica Médica Belo Horizonte MG Brazil Universidade Federal de Minas Gerais. Faculdade de Medicina. Departamento de Clínica Médica. Belo Horizonte, MG, Brazil; III Secretaria Municipal de Saúde Uberlândia MG Brasil Secretaria Municipal de Saúde. Uberlândia, MG, Brasil; IV Universidade de São Paulo Escola de Enfermagem de Ribeirão Preto Ribeirão Preto SP Brasil Universidade de São Paulo. Escola de Enfermagem de Ribeirão Preto. Ribeirão Preto, SP, Brasil; V Pontifica Universidade Católica de Minas Gerais Programa de Pós-Graduação em Geografia Belo Horizonte MG Brazil Pontifica Universidade Católica de Minas Gerais. Programa de Pós-Graduação em Geografia. Belo Horizonte, MG, Brazil; VI Secretaria Municipal de Saúde Coordenação de Saúde do Idoso Uberlândia MG Brasil Secretaria Municipal de Saúde. Coordenação de Saúde do Idoso. Uberlândia, MG, Brasil

**Keywords:** Health of the Elderly, Frailty, Primary Health Care, Public Health

## Abstract

**OBJECTIVE:**

To describe the functional clinical profile of elderly people linked to primary health care, using the Functional Clinical Vulnerability Index (IVCF-20) and to spatialize those with the greatest functional decline by primary health care units in the municipality of Uberlândia, in the state of Minas Gerais (MG), in the year 2022.

**METHODS:**

A cross-sectional study with secondary data from the Municipal Health Department of Uberlândia-MG. The variables were compared using Student’s t-test, Mann Whitney test, Pearson’s chi-square, and multinomial logistic regression to obtain the independent effect of each variable. The significance level adopted was 5% (p < 0.05). The georeferenced database in ArcGIS® was used.

**RESULTS:**

47,182 older adults were evaluated with a mean age of 70.3 years (60 to 113 years), 27,138 of whom were women (57.52%), with a clear predominance of low-risk or robust older adults (69.40%). However, 11.09% are high-risk older adults and 19.52% are at risk of frailty. Older men had independently lower odds of moderate and high risk compared to older women (OR = 0.53; p < 0.001). A high prevalence of polypharmacy was observed, 21.40% of the older adult population, particularly in frail older adults, with a prevalence of 63.08%. There was a greater distribution of frail older adults around the central region of the municipality and in health units with a larger coverage area. The IVCF-20 made it possible to screen frailty in primary health care.

**CONCLUSION:**

The instrument is capable of stratifying the risk of older adults in health care networks through primary health care, enabling the application of individualized preventive, promotional, palliative, or rehabilitative interventions, according to the clinical functional stratum of the older adult and the compromised functional domains. Risk stratification and spatial distribution of the frailest older adults can be a good strategy for qualifying health professionals with the aim of maximizing the autonomy and independence of the older adults.

## INTRODUCTION

The aging of the population is associated with a greater risk of adverse outcomes, such as a higher prevalence of chronic and acute health conditions, functional dependence, hospitalization, institutionalization and death^1^. This greater vulnerability of older adults to illness and functional decline is known as frailty^2^. Various ways of operationalizing the concept of frailty are recognized, making it difficult to apply it in clinical practice and, consequently, to replicate and compare the various existing models in population-based studies^5,6^. The current consensus is that frailty is a multidimensional geriatric syndrome, with a dynamic character, associated with the reduction of homeostatic reserves, which progressively limits the ability to resist potential acute aggressors, causing cumulative and self-perpetuating deficits, culminating in functional decline, hospitalization, institutionalization and death^7,8^.

The variability in the concept of “frail older adults” is directly reflected in the use of different diagnostic instruments^9,10^. In order to standardize frailty risk stratification in the Brazilian older adult population and the recognition of the “frail older adult” by primary health care (PHC), the National Council of Health Secretaries (CONASS), in partnership with the Ministry of Health and the *Sociedade Beneficente Israelita Brasileira* (Hospital Albert Einstein), published a technical note suggesting the use of the Functional Clinical Vulnerability Index Assessment Instrument, IVCF-20^a^. The choice of IVCF-20 was based on its simplicity and quick application, and can be used by any health professional, including community health workers^11^. The instrument consists of 20 questions that assess the main functional clinical determinants of older adult health, with scores ranging from 0 to 40 points. The higher the value obtained, the greater the risk of clinical-functional vulnerability^12^. According to Faller et al.^9^, the IVCF-20 was recognized as one of the four best instruments in the world capable of recognizing frailty in the older adult population.

With an agreement signed with CONASS, the municipality of Uberlândia has acted as a collaborating center for *Planificação da Atenção à Saúde* (PAS – Health Care Planning) since 2017. With an estimated population of 706,597 inhabitants, of whom 108,793 are older adults (15.39%)^16^, as of December 2018, the organization of the *Rede de Atenção da Pessoa Idosa* (RASPI –Care Network of Older Adults) began under the PAS perspective. RASPI was organized based on the Chronic Conditions Care Model, developed to adapt to the requirements of a public and universal health care system such as SUS^17^.

The objective of this study is to evaluate the prevalence of frailty and describe the functional clinical profile of older adults living in the city of Uberlândia, identified via risk stratification carried out by IVCF-20, as well as to analyze the spatial distribution of frailty in the city by primary health units.

## METHODS

This is a cross-sectional study using secondary data, developed in the municipality of Uberlândia, located in the Triângulo Mineiro region, state of Minas Gerais.

Primary care is offered in 56 basic family health units (UBSF) that house 88 family health teams and 14 basic health units (UBS) with 78 primary care teams^18^.

The municipality’s Health Department has worked with IVCF-20 to stratify the risk of older adults and has systematically trained health professionals since 2018, with regular clinical supervision by a specialized team.

Using the IVCF20, it is possible to carry out a clinical and functional assessment of people aged 60 and over^9^, the final score is calculated from the sum of the values attributed to each response (0 or 1), with three strata being defined: robust (≤ 6 points), at risk of frailty (7 to 14 points) and frail (≥ 15 points).

Data crossing considered dimension, subdimension, questions and answers from IVCF20 used in risk stratification. Age was considered by age group.

Risk stratification data using the IVCF-20 was recorded, from 06/21/2018 to 04/30/2022, in its own electronic medical record, *Fast Medic,* used during care at health units.

Since this is a study using a secondary database, with no identification of users, other than the natural field for cross-referencing (*Cadastro de Pessoas Físicas* – CPF [the Brazilian tax identification number], processed exclusively within the database, without disclosure in the study), we considered it unnecessary to submit the project to the ethics committee, in accordance with Resolution No. 674, of May 6, 2022, in Chapter IX.

The mass of data was generated from two reports extracted from the information system, with the IVCF20 responses and the risk stratification of the older adults treated at UBSF and UBS:

- Elderly report: presents the list of all older adults registered in the older adult program and the corresponding risk stratification;
- Questionnaire report: includes users and their respective questions and answers given to IVCF20.

The reports were extracted in Excel format (@Microsoft) and included, each one, as a table in an Access database (@Microsoft), where the processing was carried out. The fields used in the study are described in Chart.

**Chart:** Relationship, description, and origin of the fields that made up the study’s data mass.

Item	Field	Description	Source report
1	User CPF	User’s CPF	Common to both reports
2	Resp. follow-up	User’s follow-up unit	Elderly Report
3	Resp. user registration	User’s registration unit	Elderly report
4	Classification	Risk stratification in the Elderly Program	Elderly report
5	Sex	User’s gender (M or F)	Elderly Report
6	Age	User’s age (age in years)	Elderly report
7	Dt. birth	User’s date of birth	Elderly Report
8	Dt. classification	Date of completion of the current risk stratification	Elderly report
9	Dt. response	Date on which the IVCF–20 questionnaire was carried out	Questionnaires Report
10	Question	Questionnaire question (one line for each question)	Questionnaires Report
11	Response	Answer to the respective questionnaire question	Questionnaires Report

CPF: *Cadastro de Pessoas Físicas*; IVCF–20: Functional Clinical Vulnerability Index; M: male; F: Female.

Initially, the records were selected using the CPF as an identifier, to link the data from the two tables, respecting the provisions of Law 13,709 of 2018^19^.

Records with the most recent responses were then selected from the questionnaire report table, as the IVCF20 may have been answered more than once, by the same individual, during the study period, as the database is cumulative.

The inclusion criteria in the study were:

- User of health units aged 60 or over who had the risk recorded in the older adult report;
- User who had answered at least 15 questions in the IVCF20 questionnaire;
- User whose CPF was provided in both reports;
- User with Dt. Classification equal to most recent Dt. response.

Statistical tests were performed using Bioestat 5.0. The sample consisted of 47,182 individuals aged 60 and over, with an estimated population of 108,793 in the same age group^16^. The chi-square test for adherence was significant (p = 0.99), indicating the representativeness of the sample.

Student’s t- and Mann Whitney tests were used for the variables age and sex; Pearson’s chi-square in the analysis of risk prevalence for each sex.

Simple multinomial logistic regression (Biostat 5.0), to obtain the independent effect, was applied to the variables age, sex, negative self-perception of health, cognition, mood and polypharmacy on the chance of occurrence in a given risk stratum, being compared with the robust stratum, which was considered the gold standard because it was the best condition, the significance level was 5% (p < 0.05).

The mapping was carried out for the 73 health units (UBS and UBSF) in the city of Uberlândia-MG. A total of 5,175 cases of frail older adults were identified, 3,555 women and 1,620 men. A georeferenced database was produced in ArcGIS®, using health units by coverage area and neighborhoods in Uberlândia as a reference. In preparing the thematic maps, we adopted the technique of proportional geometric figures, circles, directly proportional to the intensity of vulnerability risk, containing 3 classes according to the number of older adults: 1 to 10; 10 to 100; 100 to 500.

## RESULTS

Currently, around 63,784 elderly people are stratified by IVCF-20, representing 58.61% of the older adult population. After applying the selection criteria, the final sample consisted of 47,182 older adults. The prevalence of robust patients was greater than 80% among those aged 60 to 69 years, decreasing with advancing age, reaching 4.20% among patients aged 100 years or over. There was a predominance of women (27,138) compared to men (20,044) (Table 1).

**Table 1:** Distribution of the sample according to demographic and health aspects, among older adults in the city of Uberlândia (n = 47,182).

Health dimension	Robust		At risk of frailty		Frail		Total	
			
	n	%	n	%	n	%	n	%
Age years)								
60–69	20,646	80.1	3,796	14.7	1,338	5.2	25,780	100
70–79	9,758	66.1	3,353	22.7	1,648	11.2	14,759	100
80–89	2,172	39.3	1,762	31.9	1,598	28.9	5,532	100
90–99	164	15.4	287	27	613	57.6	1,064	100
≥ 100	2	4.2	10	21.3	35	74.5	47	100
Total	32,742	69.4	9,208	19.52	5,232	11.09	47,182	100
Sex								
Female	17,956	64.8	5,952	21.9	3,590	13.2	27,138	100
Male	15,146	75.6	3,256	16.2	1,642	8.2	20,044	100
Self-perceived health								
Excellent, very good, or good	27,124	83.26	3,972	43.41	1,040	19.95	32,136	68.46
Fair or poor	5,454	16.74	5,177	56.59	4,174	80.05	14,805	31.54
Daily living activities								
Functional dependence for instrumental DLAs	505	1.55	2,145	23.45	3,767	72.25	6,417	13.67
Functional dependence for basic DLAs	33	0.1	284	3.11	1,953	37.52	2,270	4.85
Cognition								
Cognitive complaints	3,678	11.32	4,227	46.09	3,730	71.63	11,635	24.83
Suspected dementia	183	0.59	889	10.12	2,350	46.33	3,422	7.6
Mood								
Suspected depression	3,946	12.24	4,991	54.8	3,813	73.72	12,750	27.4
Mobility								
Decline in reaching, grasping and pinching	618	1.91	995	10.95	1,753	33.87	3,366	7.23
Aerobic and/or muscular capacity								
Weight loss	474	1.68	808	10.24	1,020	22.49	2,302	5.65
BMI < 22	527	2.14	428	6.64	455	12.89	1,410	4.07
CC < 31 cm	430	1.78	475	7.56	576	16.73	1,481	4.38
Gait speed > 5s	729	2.99	1,376	21.49	1,918	52.98	4,023	11.69
Gait								
Gait change	915	2.84	2,686	29.61	3,860	74.6	7,461	16.06
Repeated falls	634	1.99	1,303	14.57	1,797	35.16	3,734	8.12
Continence								
Sphincter incontinence	920	2.87	1,635	18.2	2,347	45.65	4,902	10.61
Communication								
Significant vision decline	2,650	8.24	2,536	28.03	2,199	42.66	7,385	15.93
Significant hearing decline	1,062	3.33	1,492	16.61	1,644	32.12	4,198	9.13
Multiple comorbidity								
Polypathology	489	1.54	1,077	12.1	1,292	25.68	2,858	6.27
Polypharmacy	2,624	8.31	3,924	43.68	3,219	63.08	9,767	21.4
Recent hospitalization	507	1.63	944	10.83	1,106	22.19	2,557	5.72

DLAs: Daily living activities.

Ages ranged between 60 and 113 years, with a mean of 70.3 years (SD = 7.8). There was a predominance of women (57.52%) and most of the older adults were considered robust (69.40%). The prevalence of robust patients was greater than 80% among those aged 60 to 69 years, decreasing with advancing age, reaching 4.20% among patients aged 100 years or over (Table 2).

**Table 2:** Description of age and sex according to risk classification categories.

Characteristic	Risk stratification				p-value

	Low	Moderate	High	Total	
			
	n (%)	n (%)	n (%)	n (%)	
Age (range; years)					
Mean (SD)	68.5 (6.4%)	72.8 (8.3%)	77.4 (9.7%)	70.3 (7.8%)	< 0.001
Median (IQR)	67 (9%)	72 (13%)	77 (16%)	68 (11%)	< 0.001
Minimum; maximum	60; 104	60; 113	60; 109	60; 113	
Age (category; years)					
60–69	20,646 (80.1)	3,796 (14.7)	1,338 (5.2)	25,780 (100)	< 0.001
70–79	9,758 (66.1)	3,353 (22.7)	1,648 (11.2)	14,759 (100)	
80–89	2,172 (39.3)	1,762 (31.9)	1,598 (28.9)	5,532 (100)	
90–99	164 (15.4)	287 (27.0)	613 (57.6)	1,064 (100)	
≥ 100	2 (4.2)	10 (21.3)	35 (74.5)	47 (100)	
Sex					
Female	17,956 (64.8)	5,952 (21.9)	3,590 (13.2)	27,138 (100)	< 0.001
Male	15,146 (75.6)	3,256 (16.2)	1,642 (8.2)	20,044 (100)	

SD: standard deviation; IQR: interquartile range.

There was a significant correlation between age and frailty (p < 0.001), with frailty being more prevalent in women (p < 0.001). The prevalence of low risk was higher among men (75.60%), and moderate and high risk was more prevalent among women (p < 0.001) (Table 2).

The perception of fair or poor health was high, present in 31.54% of the older adult population. Dependence for instrumental and basic daily living activities (DLAs) was present in 13.67% and 4.85% of the older adults, respectively. Consistent cognitive complaints were present in 24.83% of the population, characterizing a strong suspicion of major neurocognitive disorder. Suspicion of dementia was present in 7.60% of the older adults. The prevalence of suspected mood disorders was high, 27.40% (Chart).

Repeated falls were present in 8.12% of the older adults and a high prevalence of significant gait changes was observed (16.06%). Nutritional problems were observed in 5.65% of the older adults, and a strong suspicion of sarcopenia was observed in 11.69%, due to slow gait speed, considered the main predictor of sarcopenia in older adults. Sphincter incontinence had a prevalence of 10.61%. Significant visual problems were present in 15.93% of the elderly, in addition to hearing changes (9.13%). The biggest highlight was the high prevalence of polypharmacy, present in 21.40% of the older adult population, particularly in frail older adults, 63.08% (Table 1).

Men, when compared with women, had a lower chance of moderate (OR = 0.63; 95%CI 0.60–0.66; p < 0.001) and high (OR = 0.53; 95%CI 0.50–0. 57; p < 0.001) risk. Compared to robust older adults, the results showed that negative self-perception of health had greater odds in older adults at risk of frailty (OR = 6.48; 95%CI 6.16–6.81; p < 0.001) and frail older adults (OR = 19.95; 95%CI 18.53–21.49; p < 0.001). The chance of occurrence of cognitive complaints was 6.69 (95%CI 6.34–7.06) times higher among those at risk of frailty and 19.78 (95%CI 18.45–21.20; p < 0.001) among the frail ones. The suspicion of depression was higher in older adults at risk of frailty (OR = 8.69; 95%CI 8.24–9.17; p < 0.001) and in the frail ones (OR = 20.12; 95%CI 18.75–21.59; p < 0.001). The odds of daily use of multiple medications (polypharmacy) were higher in older adults at risk of frailty (OR = 8.55; 95%CI 8.07–9.05; p < 0.001) and in the frail ones (OR = 18.84; 95%CI 17.57–20.19; p < 0.001) (Table 3).

**Table 3:** Odds ratios for frailty and risk of frailty in relation to robust individuals in the municipality of Uberlândia, in the state of Minas Gerais.

Category	Odds ratio (95%CI)	p-value
At risk of frailty (reference: robust)		
Negative self-perception of health	6.48 (6.16–6.81)	< 0.0001
Presence of cognitive complaints	6.69 (6.34–7.06)	< 0.0001
Presence of depressive complaints	8.69 (8.24–9.17)	< 0.0001
Polypharmacy	8.55 (8.07–9.05)	< 0.0001
Frail (robust reference)		
Negative self-perception of health	19.95 (18.53–21.49)	< 0.0001
Presence of cognitive complaints	19.78 (18.45–21.20)	< 0.0001
Presence of depressive complaints	20.12 (18.75–21.59)	< 0.0001
Polypharmacy	18.84 (17.57–20.19)	< 0.0001
At risk of frailty (reference: robust)		
Age (reference: 60–69 years)		
70–79	1.89 (1.79–1.99)	< 0.001
80–89	4.43 (4.12–4.76)	< 0.001
90–99	9.42 (7.74–11.5)	< 0.001
≥ 100	27.70 (6.05–127)	< 0.001
Male (reference: female)	0.63 (0.60–0.66)	< 0.001
Frail (robust reference)		
Age (reference: 60 to 69 years old)		
70–79	2.64 (2.44–2.84)	< 0.001
80–89	11.4 (10.5–12.4)	< 0.001
90–99	56.9 (47.4–68.3)	< 0.001
≥ 100	277 (66.5–1.153 )	< 0.001
Male (reference: female)	0.53 (0.50–0.57)	< 0.001

95%CI: 95% confidence interval.

The mapping identified the health units with the highest prevalence of frailty, the spatialization of the data showed that the geographic distribution and areas of concentration of frail older adults is random, with a greater concentration around the central urban area and in health units with the largest population under their care. There was a predominance of frailty in women, approximately twice as high, particularly at UBS Tocantins, UBS Brasil, UBS D. Zulmira, UBS Luizote, UBS Martins, UBS Pampulha, UBS Patrimônio, UBS Planalto, UBS Roosevelt, and UBS Tibery (Figure).

**Figure:**
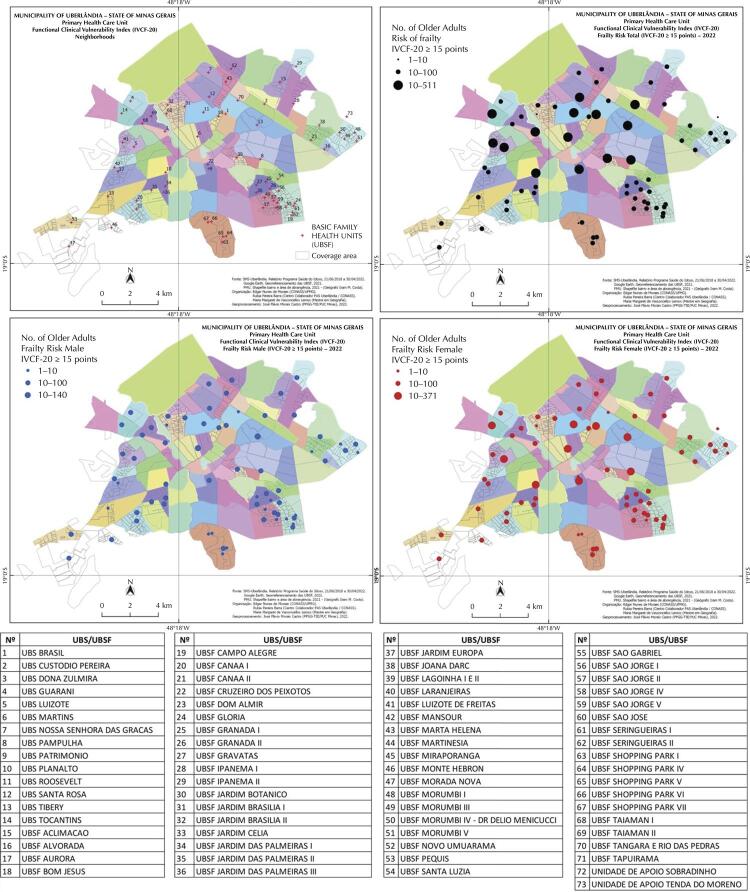
Spatialization of frail older adults, considering their basic health unit of registry and sex, in the municipality of Uberlandia, in the state of Minas Gerais

UBS: basic health units; UBSF: basic family health units

## DISCUSSION

This work is pioneering in Brazil, representing the first Brazilian population-based study that evaluated the entire older adult population of a medium-sized municipality, using the Functional Clinical Vulnerability Index-20 (IVCF-20). The prevalence of frailty was 30.61%, (IVCF-20 ≥ 7 points), while older adults with high clinical functional vulnerability (IVCF ≥ 15 points) were present in 11.09%. Other studies using the same instrument demonstrated a variation of 12% to 20% in high-risk older adults^20^.

Most older adults have low clinical and functional vulnerability—according to several authors^7^and Moraes^24^—whose health management can be carried out by PHC, following the principles of the risk pyramid model, recommended^25^, in Chronic Condition Care Model^10^.

Studies carried out on elderly people demonstrate a prevalence of robust, pre-frail and frail older adults of, respectively, 45%, 49.9%, and 5.2% using the criteria proposed by Fried et al.^26^

These variations can be explained due to the influence of demographic, social and economic factors, as well as access, use and care structure of health services^14^. Research that compared Uberlândia with two other Brazilian municipalities showed a high Human Development Index (HDI) and life expectancy^27^.

The variables related to the highest frequency of frailty were age and female gender. Other studies obtained the same finding, which can be explained by the lower concentration of lean mass and muscle strength in elderly women compared to older men^7,8,10^.

Negative self-perception of health was present in a third of the older adults and showed a dose-response gradient with the IVCF-20 score, confirming its importance as a marker of frailty. This finding was present in, respectively, 42.6%, 42.4%, 30.3%, and 28.8% of several studies^5^, reinforcing the importance of this marker of quality of life in older adults. However, another study showed 70% of negative self-perception. Notably, this finding is mainly associated with loss of autonomy and decline in functionality^15^. Another explanation is the difference in socioeconomic and demographic response options in each location. Research carried out demonstrated the association of the prevalence of negative self-rated health with worse indicators of income, education, and consumption classes^28^. Health perception, despite being a subjective measure, is considered a good indicator of the health status of older adults and may indicate the need for general health surveillance and implementation of public policies to improve quality of life.

The prevalence of functional dependence in at least one instrumental DLA (shopping, controlling finances and carrying out small household work) was 13.67%, while dependence for bathing was 4.85%. Research carried out using the same instrument demonstrated the prevalence of dependence for some DLA ranging from 55.9% to 21.6%. This variation can be explained by the region of the country and measurement methodologies. In relation to basic DLA (stopped taking a shower alone), similar studies showed little variation, corroborating our results^14,20^.

Cognitive capacity is one of the domains of intrinsic capacity and must be routinely assessed by PHC, according to the Integrated Care for Older People (ICOPE)^29^. There was a high prevalence of cognitive complaints in the older adults evaluated and the suspicion of major neurocognitive disorder was 7.6%, which coincides with the study, in which the prevalence of dementia in Brazil was 7.1%^30^.

In turn, the prevalence of suspected depression was high (27.4%), in one study, a prevalence of depression was observed in 14.5% of older adults^31^. Another relevant finding of our study was the high correlation between suspected depression and an increase in the IVCF-20 score, confirming the high association between these two chronic health conditions.

Evidence of malnutrition and slow gait speed were observed in, respectively, 5% and 11.69% of older adults in Uberlândia. Both criteria are highly suggestive of the presence of sarcopenia. Weight loss, low BMI, calf circumference less than 31 cm and, mainly, slow gait are suggested instruments for early diagnosis^32^. The prevalence of sarcopenia is also heterogeneous, depending on the diagnostic criteria used, ranging from 10 to 27% of older adults. We observed the prevalence of postural instability (presence of some difficulty in walking that could prevent the performance of some daily activity) and repeated falls in, respectively, 16.06% and 8.12% of the older adults evaluated. Carneiro et al.^15^found a higher prevalence of repeated falls (27.9%), as well as limitations in walking (27.7%), probably attributed to the study design. The World Falls Guidelines (WFG) Task Force, recently published^33^, reinforces the importance of assessing gait in older adults and the presence of falls. In our study, the presence of significant decline in vision and hearing was, respectively, 15.93% and 9.13%, lower than what was observed by Carneiro et al.^15^, which was around 20% for both.

The high prevalence of polypharmacy (use of five or more medications per day) should be highlighted in our study, noticed in 21.4% of older adults and was highly correlated with the IVCF-20 score (dose-response gradient). Polypharmacy is the main risk factor for inappropriate prescription among older adults, present in 20 to 79%, depending on the criteria used, and is therefore quite common in clinical practice^25^and is considered an important marker of healthcare costs^3 4^.

The mapping of frail older adults allowed the situational diagnosis of the spatial distribution in the municipality, with the identification of the UBS with the highest concentration. The distribution was heterogeneous, with a greater concentration around the central area of the city, probably due to the larger population assigned to the UBS and because they are older neighborhoods. Another relevant point was the greater tendency for frailty to affect females, confirming the literature. This distribution pattern was similar to that observed by Freitas et al.^21^, who carried out similar work in the municipality of Pombal, Paraíba.

The study has limitations, such as the use of a questionnaire based on self-reported responses, which may suffer from memory bias. However, all answers were confirmed by the older adult’s companion. The IVCF-20 does not replace the comprehensive geriatric assessment, considered the gold standard for geriatric-gerontological diagnosis^35^. Finally, risk stratification was carried out in 58.61% of the older adult population and around 30% of the IVCF-20 evaluated were excluded, due to the presence of inconsistency in the results.

## CONCLUSION

Risk stratification using the IVCF-20 allows screening of the clinical and functional vulnerability of the older adult population and understanding their main demands, facilitating the development of more specific guidelines and public policies. It also allows the definition of flows and counter-flows in the health care network, with a clearer definition of referral criteria for specialized outpatient care and its sizing. Risk stratification and spatial distribution of the frailest older adults can be a good strategy for qualifying health professionals with the aim of maximizing the autonomy and independence of the elderly.

## Footnotes

a Available from: http://www.ivcf20.org
