# Supplementary material for: Frailty and spatialization of older adults in the city of Uberlândia with IVCF-20
Source: Rev Saude Publica. 2024 Apr 1;57(Suppl 3):9s. doi: 10.11606/s1518-8787.2023057005273 (PMC11037899; doi:10.11606/s1518-8787.2023057005273)
Supplement: Translation

# Translation: Fragilidade e espacialização de pessoas idosas do município de Uberlândia com IVCF-20

**Authors:** Rubia Pereira Barra, Edgar Nunes de Moraes, Maria Margaret de Vasconcellos Lemos, Poliana Castro de Resende Bonati, José Flávio Morais Castro, André Augusto Jardim

**Affiliations:** I Brasil Centro Colaborador Planificação da Atenção à Saúde Uberlândia. Conselho Nacional de Secretários de Saúde. Uberlândia, MG, Brasil; II Brasil Universidade Federal de Minas Gerais. Faculdade de Medicina. Departamento de Clínica Médica. Belo Horizonte, MG, Brasil; III Brasil Secretaria Municipal de Saúde. Uberlândia, MG, Brasil; IV Brasil Universidade de São Paulo. Escola de Enfermagem de Ribeirão Preto. Ribeirão Preto, SP, Brasil; V Brasil Pontifica Universidade Católica de Minas Gerais. Programa de Pós-Graduação em Geografia. Belo Horizonte, MG, Brasil; VI Brasil Secretaria Municipal de Saúde. Coordenação de Saúde do Idoso. Uberlândia, MG, Brasil

**Keywords:** Saúde do Idoso, Fragilidade, Atenção Primária à Saúde, Saúde Pública

## Abstract

**OBJETIVO:**

Descrever o perfil clínico funcional dos idosos vinculados à atenção primária à saúde, por meio do Índice de Vulnerabilidade Clínico Funcional (IVCF-20) e realizar a espacialização dos de maior declínio funcional por unidades de atenção primária à saúde no município de Uberlândia-MG, no ano de 2022.

**MÉTODOS:**

Estudo transversal com dados secundários da Secretaria Municipal de Saúde de Uberlândia-MG. As variáveis foram comparadas através de testes de t de Student, Mann Whitney, qui-quadrado de Pearson e regressão logística multinomial para obter o efeito independente de cada variável. O nível de significância adotado foi de 5% (p < 0,05). Utilizou-se o banco de dados georreferenciado no ArcGIS®.

**RESULTADOS:**

Foram avaliados 47.182 idosos com idade média de 70,3 anos (60 a 113 anos), sendo 27.138 mulheres (57,52%) e observou-se franco predomínio de idosos de baixo risco ou robustos (69,40%). Todavia, 11,09% são idosos de alto risco e 19,52% estão em risco de fragilização. Idosos do sexo masculino tiveram chance independentemente inferior de risco moderado e alto comparados às mulheres idosas (OR = 0,53; p < 0,001). Foi observado alta prevalência de polifarmácia, 21,40% da população idosa, particularmente nos idosos frágeis, com prevalência de 63,08%. Houve maior distribuição dos idosos frágeis em torno da região central do município e nas unidades de saúde com maior área de abrangência. O IVCF-20 permitiu realizar o rastreio da fragilidade na atenção primária à saúde.

**CONCLUSÃO:**

O instrumento é capaz de estratificar o risco dos idosos nas redes de atenção à saúde pela atenção primária à saúde, possibilitando a aplicação de intervenções preventivas, promocionais, paliativas ou reabilitadoras individualizadas, conforme o estrato clínico funcional do idoso e os domínios funcionais comprometidos. A estratificação de risco e a distribuição espacial dos idosos mais frágeis pode ser uma boa estratégia de qualificação dos profissionais de saúde com o objetivo de maximizar a autonomia e independência dos idosos.

## INTRODUÇÃO

O envelhecimento da população está associado a maior risco de desfechos adversos, como maior prevalência de condições crônicas e agudas de saúde, dependência funcional, hospitalização, institucionalização e óbito^1^. Essa maior vulnerabilidade da pessoa idosa ao adoecimento e ao declínio funcional é conhecido como fragilidade^2^. Diversas formas de operacionalização do conceito de fragilidade são reconhecidas, dificultando sua aplicação na prática clínica e, consequentemente, a replicação e comparação dos diversos modelos existentes em estudos de base populacional^5,6^. O consenso atual é que a fragilidade é uma síndrome geriátrica multidimensional, com caráter dinâmico, associada à redução das reservas homeostáticas, que limita progressivamente a capacidade de resistir a potenciais agressores agudos, provocando déficits cumulativos e auto perpetuantes, culminando com o declínio funcional, internação, institucionalização e óbito^7,8^.

A variabilidade no conceito de “idoso frágil” reflete-se diretamente na utilização de diversos instrumentos diagnósticos^9,10^. No intuito de padronizar a estratificação de risco de fragilidade na população idosa brasileira e o reconhecimento do “idoso frágil” pela atenção primária à saúde (APS), o Conselho Nacional de Secretários de Saúde (CONASS), em parceria com o Ministério da Saúde e Sociedade Beneficente Israelita Brasileira (Hospital Albert Einstein), publicou nota técnica sugerindo a utilização do Instrumento de Avaliação do Índice de Vulnerabilidade Clínico Funcional, IVCF-20^a^. A escolha do IVCF-20 baseou-se na sua simplicidade e rápida aplicação, podendo ser utilizado por qualquer profissional de saúde, incluindo o agente comunitário de saúde^11^. O instrumento é composto por 20 questões que avaliam os principais determinantes clínicos funcionais da saúde do idoso, cuja pontuação varia de 0 a 40 pontos. Quanto mais alto o valor obtido, maior o risco de vulnerabilidade clínico-funcional^12^. Segundo Faller et al.^9^, o IVCF-20 foi reconhecido como sendo um dos quatro melhores instrumentos do mundo capazes de reconhecer a fragilidade na população idosa.

Por meio de convênio firmado com o CONASS, o município de Uberlândia atua como Centro Colaborador da Planificação da Atenção à Saúde (PAS) desde 2017. Com uma população estimada de 706.597 habitantes, dos quais 108.793 são idosos (15,39%)^16^, a partir de dezembro de 2018 iniciou a organização da Rede de Atenção da Pessoa Idosa (RASPI) sob a ótica da PAS. A organização da RASPI se deu a partir do Modelo de Atenção as Condições Crônicas (MACC), desenvolvido para se adaptar as exigências de um sistema de atenção à saúde público e universal como o SUS^17^.

O objetivo deste estudo é avaliar a prevalência de fragilidade e descrever o perfil clínico funcional dos idosos residentes no município de Uberlândia, identificados por meio da estratificação de risco realizada pelo IVCF-20, assim como analisar a distribuição espacial da fragilidade no município por unidades de atenção primária.

## MÉTODOS

Trata-se de um estudo transversal com o emprego de dados secundários, desenvolvido no município de Uberlândia, localizado na região do Triângulo Mineiro, estado de Minas Gerais.

A atenção primária é oferecida em 56 unidades básicas de saúde da família (UBSF) que abrigam 88 equipes de saúde da família (ESF) e 14 unidades básicas de saúde (UBS) com 78 equipes de atenção primária^18^.

A Secretaria de Saúde do município, tem trabalhado com o IVCF-20 para a estratificação de risco dos idosos e capacita, sistematicamente, profissionais de saúde, desde 2018, com supervisão clínica regular, por equipe especializada.

Através do IVCF20 é possível fazer uma avaliação clínica e funcional das pessoas com 60 anos e mais^9^, o escore final é calculado a partir soma dos valores atribuídos a cada resposta (0 ou 1), sendo definidos três estratos: robustos (< 6 pontos), em risco de fragilização (7 a 14 pontos) e frágeis (≥ 15 pontos).

O cruzamento dos dados considerou dimensão, subdimensão, perguntas e respostas do IVCF20 empregadas na estratificação de risco. A idade foi trabalhada por faixa etária.

Foram trabalhados dados de estratificação de risco pelo IVCF-20 registrados, de 21/06/2018 a 30/04/2022, em prontuário eletrônico próprio, *Fast Medic,* usado durante os atendimentos nas unidades de saúde.

Por ser se tratar de estudo utilizando base de dados secundários, sem identificação dos usuários, que não seja o campo natural para cruzamento (Cadastro de Pessoas Físicas (CPF), tratado, exclusivamente, dentro do banco de dados, sem divulgação no estudo), consideramos ser dispensável a submissão do projeto para comitê de ética, conforme a Resolução Nº 674, de 6 de maio de 2022, no Capítulo IX.

A massa de dados foi gerada a partir de dois relatórios extraídos do sistema de informação, com as respostas do IVCF20 e a estratificação de risco dos idosos atendidos nas UBSF e UBS:

- Relatório de idosos: apresenta a relação de todos os idosos cadastrados no programa de idoso e a correspondente estratificação de risco;
- Relatório de questionários: inclui os usuários e as respectivas perguntas e respostas dadas ao IVCF20.

Os relatórios foram extraídos no formato Excel (@Microsoft) e incluídos, cada um, como tabela em um banco de dados do Access (@Microsoft), onde foi realizado o processamento, os campos usados no estudo estão descritos no Quadro.

**Quadro:** Relação, descrição e origem dos campos que compuseram a massa de dados do estudo.

Item	Campo	Descrição	Relatório de origem
1	CPF usuário	CPF do usuário	Comum aos dois relatórios
2	Resp. acompanhamento	Unidade de acompanhamento do usuário	Relatório de idosos
3	Resp. cadastro usuário	Unidade de cadastro do usuário	Relatório de idosos
4	Classificação	Estratificação do risco no Programa do Idoso	Relatório de idosos
5	Sexo	Sexo do usuário (M ou F)	Relatório de idosos
6	Idade	Idade do usuário (idade em anos)	Relatório de idosos
7	Dt. nascimento	Data de nascimento do usuário	Relatório de idosos
8	Dt. classificação	Data de realização da atual estratificação de risco	Relatório de idosos
9	Dt. resposta	Data em que foi realizado o questionário do IVCF–20	Relatório de questionários
10	Pergunta	Pergunta do questionário (uma linha para cada pergunta)	Relatório de questionários
11	Resposta	Resposta a respectiva pergunta do questionário	Relatório de questionários

CPF: Cadastro de Pessoas Físicas; IVCF–20: Índice de Vulnerabilidade Clínico Funcional; M: masculino; F: Feminino.

Inicialmente, selecionaram-se os registros usando-se o CPF como identificador, para lincar os dados das duas tabelas, respeitando-se o disposto na Lei 13.709 de 2018^19^.

Na sequência foram selecionados na tabela de relatórios de questionários os registros com as respostas mais recentes, pois o IVCF20 pode ter sido respondido em mais de um momento, pelo mesmo indivíduo, no período do estudo, pois a base de dados é acumulativa.

Os critérios de inclusão no estudo foram:

- Usuário das unidades de saúde com 60 anos ou mais que tivesse o risco registrado no relatório de idosos;
- Usuário que tivesse respondido no mínimo 15 perguntas do questionário do IVCF20;
- Usuário cujo CPF estivesse informado nos dois relatórios;
- Usuário com Dt. Classificação igual a Dt. Resposta mais recente.

Os testes estatísticos foram realizados pelo Bioestat 5.0. A amostra foi composta por 47.182 indivíduos de 60 anos e mais, sendo a população estimada de 108.793 da mesma faixa etária^16^. O teste de qui-quadrado para aderência foi significativo (p = 0,99), indicando que a representatividade da amostra.

Os testes t de Student e Mann Whitney foram usados para as variáveis idade e sexo; o qui-quadrado de Pearson na análise de prevalências de risco em cada sexo.

A regressão logística multinomial simples (Biostat 5.0), para obter o efeito independente, foi aplicada as variáveis idade, sexo, autopercepção negativa da saúde, cognição, humor e polifarmácia sobre a chance de ocorrência em dado estrato de risco, sendo comparadas com o estrato robusto, que por ser a melhor condição foi considerada como padrão ouro, o nível de significância foi de 5% (p < 0,05).

O mapeamento foi realizado para as 73 unidades de saúde (UBS e UBSF) da cidade de Uberlândia-MG. Foram identificados 5.175 casos de idosos frágeis, sendo 3.555 mulheres e 1.620 homens. Foi produzido um banco de dados georreferenciado no ArcGIS®, adotando-se como referência as unidades de saúde por área de abrangência e por bairros de Uberlândia. Na elaboração dos mapas temáticos foi adotada a técnica das figuras geométricas proporcionais, círculos, diretamente proporcional à intensidade de risco de vulnerabilidade, contendo 3 classes conforme o número de idosos: 1 a 10; 10 a 100; 100 a 500.

## RESULTADOS

Atualmente, cerca de 63.784 idosos encontram-se estratificados pelo IVCF-20, representando 58,61% da população idosa. Após a aplicação dos critérios de seleção, a amostra final foi de 47.182 idosos. A prevalência de robustos foi superior a 80% daqueles com 60 a 69 anos, decrescendo com o avanço da idade, chegando a 4,20% entre pacientes com 100 anos ou mais. Houve predomínio de mulheres (27.138) em relação aos homens (20.044) (Tabela 1).

**Tabela 1:** Distribuição da amostra segundo aspectos demográficos e de saúde, em idosos no município de Uberlândia (n = 47.182).

Dimensão da saúde	Robusto		Em fragilização		Frágil		Total	
			
	n	%	n	%	n	%	n	%
Idade (anos)								
60–69	20.646	80,1	3.796	14,7	1.338	5,2	25.780	100
70–79	9.758	66,1	3.353	22,7	1.648	11,2	14.759	100
80–89	2.172	39,3	1.762	31,9	1.598	28,9	5.532	100
90–99	164	15,4	287	27	613	57,6	1.064	100
≥ 100	2	4,2	10	21,3	35	74,5	47	100
Total	32.742	69,4	9.208	19,52	5.232	11,09	47.182	100
Sexo								
Feminino	17.956	64,8	5.952	21,9	3.590	13,2	27.138	100
Masculino	15.146	75,6	3.256	16,2	1.642	8,2	20.044	100
Autopercepção da saúde								
Excelente, muito boa ou boa	27.124	83,26	3.972	43,41	1.040	19,95	32.136	68,46
Regular ou ruim	5.454	16,74	5.177	56,59	4.174	80,05	14.805	31,54
Atividades de vida diária								
Dependência funcional em AVD instrumental	505	1,55	2.145	23,45	3.767	72,25	6.417	13,67
Dependência funcional em AVD básica	33	0,1	284	3,11	1.953	37,52	2.270	4,85
Cognição								
Queixas cognitivas	3.678	11,32	4.227	46,09	3.730	71,63	11.635	24,83
Suspeita de demência	183	0,59	889	10,12	2.350	46,33	3.422	7,6
Humor								
Suspeita de depressão	3.946	12,24	4.991	54,8	3.813	73,72	12.750	27,4
Mobilidade								
Declínio no alcance, preensão e pinça	618	1,91	995	10,95	1.753	33,87	3.366	7,23
Capacidade aeróbia e/ou muscular								
Perda de peso	474	1,68	808	10,24	1.020	22,49	2.302	5,65
IMC < 22	527	2,14	428	6,64	455	12,89	1.410	4,07
CP < 31 cm	430	1,78	475	7,56	576	16,73	1.481	4,38
Velocidade da marcha > 5s	729	2,99	1.376	21,49	1.918	52,98	4.023	11,69
Marcha								
Alteração da marcha	915	2,84	2.686	29,61	3.860	74,6	7.461	16,06
Quedas de repetição	634	1,99	1.303	14,57	1.797	35,16	3.734	8,12
Continência								
Incontinência esfincteriana	920	2,87	1.635	18,2	2.347	45,65	4.902	10,61
Comunicação								
Declínio significativo da visão	2.650	8,24	2.536	28,03	2.199	42,66	7.385	15,93
Declínio significativo da audição	1.062	3,33	1.492	16,61	1.644	32,12	4.198	9,13
Comorbidade múltipla								
Polipatologia	489	1,54	1.077	12,1	1.292	25,68	2.858	6,27
Polifarmácia	2.624	8,31	3.924	43,68	3.219	63,08	9.767	21,4
Internação recente	507	1,63	944	10,83	1.106	22,19	2.557	5,72

AVD: atividades da vida diária.

As idades variaram entre 60 e 113 anos, com média de 70,3 anos (DP = 7,8), Houve predomínio de mulheres, 57,52% e a maioria dos idosos foram considerados robustos, 69,40%. A prevalência de robustos foi superior a 80% daqueles com 60 a 69 anos, decrescendo com o avanço da idade, chegando a 4,20% entre pacientes com 100 anos ou mais (Tabela 2).

**Tabela 2:** Descrição de idade e sexo segundo categorias de classificação de risco.

Categoria	Estratificação do risco				valor de p

	Baixo	Moderado	Alto	Total	
			
	n (%)	n (%)	n (%)	n (%)	
Idade (intervalar; anos)					
Média (DP)	68,5 (6,4%)	72,8 (8,3%)	77,4 (9,7%)	70,3 (7,8%)	< 0,001
Mediana (IIQ)	67 (9%)	72 (13%)	77 (16%)	68 (11%)	< 0,001
Mínimo; máximo	60; 104	60; 113	60; 109	60; 113	
Idade (categoria; anos)					
60–69	20.646 (80,1)	3.796 (14,7)	1.338 (5,2)	25.780 (100)	< 0,001
70–79	9.758 (66,1)	3.353 (22,7)	1.648 (11,2)	14.759 (100)	
80–89	2.172 (39,3)	1.762 (31,9)	1.598 (28,9)	5.532 (100)	
90–99	164 (15,4)	287 (27,0)	613 (57,6)	1.064 (100)	
≥ 100	2 (4,2)	10 (21,3)	35 (74,5)	47 (100)	
Sexo					
Feminino	17.956 (64,8)	5.952 (21,9)	3.590 (13,2)	27.138 (100)	< 0,001
Masculino	15.146 (75,6)	3.256 (16,2)	1.642 (8,2)	20.044 (100)	

DP: desvio padrão; IIQ: intervalo interquartílico.

Houve correlação significativa entre idade e fragilidade (p < 0,001), sendo a fragilidade mais prevalente nas mulheres (p < 0,001). A prevalência de baixo risco foi maior entre os homens (75,60%) e moderado e alto risco foi mais prevalente entre as mulheres (p < 0,001) (Tabela 2).

A percepção da saúde regular ou ruim foi elevada, presente em 31,54% da população idosa. A dependência em atividades da vida diária (AVD) instrumental e básica estiveram presentes em 13,67% e 4,85% dos idosos, respectivamente. Queixas cognitivas consistentes foram presentes em 24,83% da população caracterizando uma forte suspeita de transtorno neuro cognitivo maior. A suspeita de demência esteve presente em 7,60% dos idosos. A prevalência de suspeita de transtorno do humor foi elevada, 27,40% (Tabela 1).

Quedas de repetição estiveram presentes em 8,12% dos idosos e foi observado alta prevalência de alterações significativas da marcha (16,06%). Problemas nutricionais foram observados em 5,65% dos idosos e forte suspeita de sarcopenia foi observada em 11,69%, pela lentificação na velocidade da marcha, considerada principal preditor de sarcopenia em idosos. A incontinência esfincteriana teve prevalência de 10,61%. Problemas visuais significativos estiveram presentes em 15,93% dos idosos, além de alterações da audição (9,13%). O destaque maior foi a alta prevalência de polifarmácia, presente em 21,40% da população idosa, particularmente nos idosos frágeis, 63,08% (Tabela 1).

Os homens quando comparados às mulheres tiveram chance inferior de risco moderado (OR = 0,63; IC95% 0,60–0,66; p < 0,001) e alto (OR = 0,53; IC95% 0,50–0,57; p < 0,001). Em comparação com idosos robustos, os resultados apontaram que autopercepção negativa da saúde teve chances maiores em idosos em risco de fragilização (OR = 6,48; IC95% 6,16–6,81; p < 0,001) e idosos frágeis (OR = 19,95; IC95% 18,53–21,49; p < 0,001). Já a chance de ocorrência de queixas cognitivas foi 6,69 (IC95% 6,34–7,06) vezes maior entre os em risco de fragilização e 19,78 (IC95% 18,45–21,20; p < 0,001) entre os frágeis. A suspeita de depressão foi maior nos idosos em fragilização (OR = 8,69; IC95% 8,24–9,17; p < 0,001) e nos frágeis (OR = 20,12; IC95% 18,75–21,59; p < 0,001). A chance de uso diário de múltiplos medicamentos (polifarmácia) foi maior nos idosos em fragilização (OR = 8,55; IC95% 8,07–9,05; p < 0,001) e nos idosos frágeis (OR = 18,84; IC95% 17,57–20,19; p < 0,001) (Tabela 3).

**Tabela 3:** Razões de chance para fragilidade e risco de fragilização em relação aos indivíduos robustos no município de Uberlândia–MG.

Categoria	Razão de chance (IC95%)	Valor de p
Em fragilização (referência robusto)		
Autopercepção negativa da saúde	6,48 (6,16–6,81)	< 0,0001
Presença de queixas cognitivas	6,69 (6,34–7,06)	< 0,0001
Presença de queixas depressivas	8,69 (8,24–9,17)	< 0,0001
Polifarmácia	8,55 (8,07–9,05)	< 0,0001
Frágil (referência robusto)		
Autopercepção negativa da saúde	19,95 (18,53–21,49)	< 0,0001
Presença de queixas cognitivas	19,78 (18,45–21,20)	< 0,0001
Presença de queixas depressivas	20,12 (18,75–21,59)	< 0,0001
Polifarmácia	18,84 (17,57–20,19)	< 0,0001
Em fragilização (referência robusto)		
Idade (referência: 60–69 anos)		
70–79	1,89 (1,79–1,99)	< 0,001
80–89	4,43 (4,12–4,76)	< 0,001
90–99	9,42 (7,74–11,5)	< 0,001
≥ 100	27,70 (6,05–127)	< 0,001
Sexo masculino (referência: sexo feminino)	0,63 (0,60–0,66)	< 0,001
Frágil (referência robusto)		
Idade (referência: 60 a 69 anos)		
70–79	2,64 (2,44–2,84)	< 0,001
80–89	11,4 (10,5–12,4)	< 0,001
90–99	56,9 (47,4–68,3)	< 0,001
≥ 100	277 (66,5–1.153 )	< 0,001
Sexo masculino (referência: sexo feminino)	0,53 (0,50–0,57)	< 0,001

IC95%: intervalo de confiança de 95%.

O mapeamento identificou as unidades de saúde com maior prevalência de fragilidade, a espacialização dos dados evidenciou que a distribuição geográfica e as áreas de concentração dos idosos frágeis é aleatória, com maior concentração em torno da área central urbana e nas unidades de saúde com maior população adscrita. Observou-se o predomínio de fragilidade nas mulheres, cerca de duas vezes superior, particularmente nas UBS Tocantins, UBS Brasil, UBS D. Zulmira, UBS Luizote, UBS Martins, UBS Pampulha, UBS Patrimônio, UBS Planalto, UBS Roosevelt e UBS Tibery (Figura).

**Figura:** Espacialização dos idosos frágeis, considerando-se a unidade de atenção primaria de cadastro e o sexo, no município de Uberlândia-MG.

UBS: unidades básicas de saúde; UBSF: unidades básicas de saúde da família.

## DISCUSSÃO

Este trabalho é pioneiro no Brasil, representa o primeiro estudo brasileiro de base populacional que avaliou toda a população idosa de um município de médio porte, utilizando o Índice de Vulnerabilidade Clínico Funcional-20 (IVCF-20). A prevalência de fragilidade foi de 30,61%, (IVCF-20 ≥ 7pontos), enquanto idosos com alta vulnerabilidade clínico funcional (IVCF ≥ 15 pontos), esteve presente em 11,09%. Outros estudos utilizando o mesmo instrumento demonstraram variação de 12% a 20% em idosos de alto risco^20^.

A maioria dos idosos é de baixa vulnerabilidade clínico funcional, conforme vários autores^7^ e Moraes^24^, cuja gestão da saúde pode ser realizada pela APS, seguindo os princípios do modelo da pirâmide de riscos, preconizado^25^, no MACC^10^.

Estudos realizados em idosos demonstram prevalência de idosos robustos, pré-frágeis e frágeis de, respectivamente, 45%, 49,9% e 5,2% utilizando-se os critérios propostos por Fried et al.^26^.

Essas variações podem ser explicadas devido a influência de fatores demográficos, sociais, econômicos, bem como o acesso, utilização e estrutura assistencial dos serviços de saúde^14^. Pesquisa que comparou Uberlândia com mais dois municípios brasileiros evidenciou alto Índice de Desenvolvimento Humano (IDH) e expectativa de vida^27^.

As variáveis relacionadas com maior frequência de fragilidade foram idade e sexo feminino. Outros estudos obtiveram o mesmo achado, o que pode ser explicado pela menor concentração de massa magra e força muscular nas mulheres idosas em relação aos homens idosos^7,8,10^.

Autopercepção negativa da saúde esteve presente em um terço dos idosos e apresentou gradiente dose-resposta com a pontuação no IVCF-20, confirmando sua importância como marcador de fragilidade. Este achado esteve presente em, respectivamente, 42,6%, 42,4%, 30,3% e 28,8% de vários estudos^5^ reforçando a importância desse marcador de qualidade de vida no idoso. Entretanto outro estudo evidenciou 70% de autopercepção negativa. Ressalta-se que esse achado está associado principalmente a perda da autonomia e declínio de funcionalidade^15^. Outra explicação é a diferença de opções de respostas, socioeconômicas e demográficas de cada local. Pesquisa realizada demonstrou a associação da prevalência de autoavaliação negativa de saúde com piores indicadores de renda, escolaridade e classes de consumo^28^. A percepção de saúde apesar de ser uma medida subjetiva é considerada como bom indicador do estado de saúde dos idosos, pode indicar necessidade de vigilância da saúde geral e efetivação das políticas públicas para melhoria da qualidade de vida.

A prevalência de dependência funcional em pelo menos uma AVD instrumental (fazer compras, controlar finanças e realizar pequenos trabalhos domésticos) foi de 13,67%, enquanto a dependência para o banho foi de 4,85%. Pesquisas realizadas utilizando mesmo instrumento demonstraram a prevalência de dependência em alguma AVD variando de até 55,9% a 21,6%. Essa variação pode ser explicada pela região do país e metodologias para mensuração. Em relação às AVD básicas (deixaram de tomar banho sozinhos) estudos semelhantes evidenciaram pouca variação corroborando com os nossos resultados^14,20^.

A capacidade cognitiva é um dos domínios da capacidade intrínseca e deve ser avaliada rotineiramente pela APS, segundo o Programa de Atenção Integrada para a Pessoa Idosa (ICOPE)^29^. Houve alta prevalência de queixas cognitivas nos idosos avaliados e a suspeita de transtorno neurocognitivo maior foi de 7,6%, que coincide com o estudo, no qual a prevalência de demência no Brasil foi de 7,1%^30^.

Por sua vez, a prevalência de suspeita de depressão foi elevada (27,4%), em um estudo, observou-se uma prevalência de depressão em 14,5% dos idosos^31^. Outro achado relevante do nosso estudo foi a alta correlação entre suspeita de depressão e aumento na pontuação do IVCF-20, confirmando a alta associação entre essas duas condições crônicas de saúde.

Evidências de subnutrição e lentificação da velocidade da marcha foram observados em, respectivamente, 5% e 11,69% dos idosos de Uberlândia. Ambos os critérios são altamente sugestivos da presença de sarcopenia. Perda de peso, baixo IMC, circunferência da panturrilha menor que 31 cm e, principalmente, lentificação da marcha são instrumentos sugeridos para o diagnóstico precoce^32^. A prevalência de sarcopenia é também heterogênea, dependendo dos critérios diagnósticos utilizados, variando de 10 a 27% dos idosos. Observamos a prevalência de instabilidade postural (presença de alguma dificuldade para caminhar capaz de impedir a realização de alguma atividade do cotidiano) e de quedas de repetição em, respectivamente, 16,06% e 8,12% dos idosos avaliados. Carneiro et al.^15^ encontraram prevalência de quedas de repetição mais elevada (27,9%), assim como de limitação para caminhar (27,7%), provavelmente atribuídas ao desenho do estudo. O *World Falls Guidelines* (WFG) *Task Force*, publicado recentemente^33^, reforça a importância da avaliação da marcha no idoso e da presença de quedas. No nosso estudo, a presença de declínio significativo da visão e da audição foi de, respectivamente, 15,93% e 9,13%, mais baixas do que foi observado por Carneiro et al.^15^, que foi de cerca de 20% para ambas.

A alta prevalência de polifarmácia (uso de cinco ou mais medicamentos por dia) deve ser destacada no nosso estudo, percebido em 21,4% dos idosos e foi altamente correlacionada com a pontuação no IVCF-20 (gradiente dose-resposta). A polifarmácia é o principal fator de risco para a prescrição inapropriada em idosos, presente em 20 a 79%, dependendo do critério utilizado, sendo, portanto, bastante frequente na prática clínica^25^ e é considerado importante marcador dos custos assistenciais^34^.

O mapeamento dos idosos frágeis permitiu o diagnóstico situacional da distribuição espacial no município, com a identificação das UBS com maior concentração. A distribuição foi heterogênea, com maior concentração em torno da área central da cidade, provavelmente pela maior população adscrita às UBS e por serem bairros mais antigos. Outro ponto relevante foi a maior tendência da fragilidade no sexo feminino, confirmando os dados da literatura. Este padrão de distribuição foi semelhante ao observado por Freitas et al.^21^, que realizou trabalho semelhante no município de Pombal, Paraíba.

O estudo apresenta limitações, como a utilização de um questionário baseado em respostas autorreferidas, que podem sofrer viés de memória. Todavia, todas as respostas foram confirmadas pelo acompanhante da pessoa idosa. O IVCF-20 não substitui a avaliação geriátrica ampla, considerada o padrão ouro para o diagnóstico geriátrico-gerontológico^35^. Por fim, a estratificação de risco foi realizada em 58,61% da população idosa e foram excluídos cerca de 30% dos IVCF-20 avaliados, pela presença de inconsistência nos resultados.

## CONCLUSÃO

A estratificação de risco pelo IVCF-20 permite realizar o rastreio da vulnerabilidade clínico funcional da população idosa e conhecer as suas principais demandas, facilitando o desenvolvimento de diretrizes e políticas públicas mais específicas. Permite também a definição de fluxos e contrafluxos na rede de atenção à saúde, com a definição mais clara de critérios de encaminhamento para a atenção ambulatorial especializada e do seu dimensionamento. A estratificação de risco e a distribuição espacial dos idosos mais frágeis pode ser uma boa estratégia para qualificação dos profissionais de saúde com o objetivo de maximizar a autonomia e independência dos idosos.

## Footnotes

a Disponível em: http://www.ivcf20.org
